# An assessment of the spatial extent of polar dust using satellite thermal data

**DOI:** 10.1038/s41598-020-79825-7

**Published:** 2021-01-13

**Authors:** M. Bowen, R. F. Vincent

**Affiliations:** Department of Physics and Space Science, Royal Military College of Canada, Kingston, K7K 7B4 Canada

**Keywords:** Environmental impact, Cryospheric science

## Abstract

The effect of mineral dust aerosols and subsequent deposition in polar regions has historically been underestimated in climate models. Dust aerosols increase melt rates by reducing surface albedo and modifying atmospheric radiative properties. In this study 127,413 satellite images covering the Arctic and Antarctic from 2007 to 2019 were assessed for dust content using thermal infrared wavelengths. The results show a strong linear trend in which the relative spatial extent of dust (RSED) increased annually by 0.31% in the Arctic (8.5% to 12.1%) and 0.19% in the Antarctic (5.2% to 7.5%). Seasonally, the maximum aggregated average RSED occurred in the Arctic during boreal winter (11.2%), while the Antarctic peaked in austral spring (9.5%). Maximum RSED rates occurred in boreal winter/austral summer (Dec–Jan–Feb) for both polar regions. The data suggests that finer dust particles are more efficiently distributed by aeolian processes leading to higher RSED values that are not necessarily reflective of polar dust loading models.

## Introduction

Dust particles affect the environment by modifying cloud behavior, disrupting solar and terrestrial radiation patterns, and altering surface properties^1–3^. Changes in climate are most acutely manifested in polar regions, a process known as polar amplification^4^, particularly in the Arctic where surface temperatures are increasing faster than anywhere else on Earth^5,6^. The deposition of light absorbing particulates onto snow and ice surfaces in high latitudes reduces albedo and leads to accelerated melt rates^7,8^, while aerosols impact normal cloud radiative properties by altering ice nucleation rates^9^.

Atmospheric aerosols consist of non-mineral and mineral particles. Non-mineral dust aerosols include naturally occurring black carbon and sulfuric acid produced by volcanic eruptions and forest fires, as well as black carbon, sulphates, and nitrates from anthropogenic sources^11,12^. Mineral dust aerosols, hereafter referred to as ‘dust’, mirrors the elemental composition of the upper continental crust with silicates such as feldspar and quartz dominating^13^. Aeolian processes preferentially remove fine particles from the source and may cause further particle fractionation, leading to particle size disparity between source material and aerosol^13^. Dust transported by wind varies between 2 and 125 µm in diameter, although the majority is comprised of silts ranging from 10 µm to 50 µm^14^.

The rate of dust deposition at the poles is greater than the global average^15^. Approximately 6.5 million metric tons of dust are deposited yearly between 60° N and 90° N latitude^16^ with that figure expected to rise as desertification and aridity increase globally^17^. Mineral dust in the Arctic is sourced from Asia (38%), Africa (32%), and locally (27%)^16,18^. In situ dust is primarily created from retreating glaciers, exposing fine sediments that are entrained by aeolian processes^19^. A dust belt from northwest Africa to East Asia contributes up to 75% of all global mineral dust aerosols^20^ with the Gobi and Taklamakan deserts contributing to Arctic dust concentrations more strongly than the Sahara^2,21^. Evidence suggests that dust is transported directly to the Arctic in 25% of Asian dust storms, which peaks during boreal spring^22^. In the southern polar region, dust originates primarily from southern Oceania, the Patagonia region of South America, and to a lesser extent, South Africa^23^. The Antarctic coast consists of 93% marine-terminating ice^24^, so local dust generation is minimal outside of the McMurdo Dry Valleys and ancient supraglacial debris bands^25,26^. About 0.6 million metric tons of dust are deposited yearly between 60° S and 90° S latitude^16^, with South America (~ 50%), Australia (~ 33%) and New Zealand (~ 14%) as the primary sources^23,27^. The deposition of dust in the Antarctic is not homogeneous, with the Atlantic and Western sectors of Antarctica dominated by emissions from Patagonia and New Zealand, while Australian and South African dust is more prevalent in Eastern Antarctica^27^. There is less dust transported to the Antarctic than the Arctic because of less favorable wind patterns and a smaller amount of landmass in the Southern Hemisphere.

The temporal distribution, physical extent, and environmental effect of mineral dust at high latitudes is an underrepresented facet of polar climate science and the impact of dust deposition on cryospheric processes is not well understood^10,28^. This research adds to the growing body of knowledge on polar dust loading by measuring the relative spatial extent of dust (RSED) in the Arctic and Antarctic from 2007 to 2019 using satellite thermal infrared (TIR) imagery. The 12-year record of RSED establishes annual, seasonal and monthly trends in areal dust distribution at the poles.

## Methods

The detection of dust using satellite thermal data is well established in the scientific community^29–34^. Numerous studies have shown that dust may be detected with a spaceborne radiometer by comparing bands in the TIR regime. The selective emissivity of snow/ice, water, vegetation, and sand allows these materials to be identified by measuring the brightness temperature difference between 11 μm and 12 μm (BTD_11–12_). BTD_11–12_ is highly positive for snow/ice, slightly positive for water, slightly negative for vegetation and highly negative for sand or dust^29,35^. A problem with identifying dust with BTD_11-12_ is discriminating it from the contribution of various surface types^30^. This issue is mitigated in the polar regions where ice/snow and water predominate. Arctic tundra has been noted to have a highly positive BTD_11–12_ signature^30^, which means that both poles generally exhibit a positive surface BTD_11–12_ value throughout the year. The predominantly positive BTD_11-12_ signature at high latitudes allows the identification of dust with Advanced Very High Resolution Radiometry (AVHRR) satellite data by applying a threshold of BTD_11-12_ < 0 K to each pixel in a polar scene^30^.

**Figure 1:**
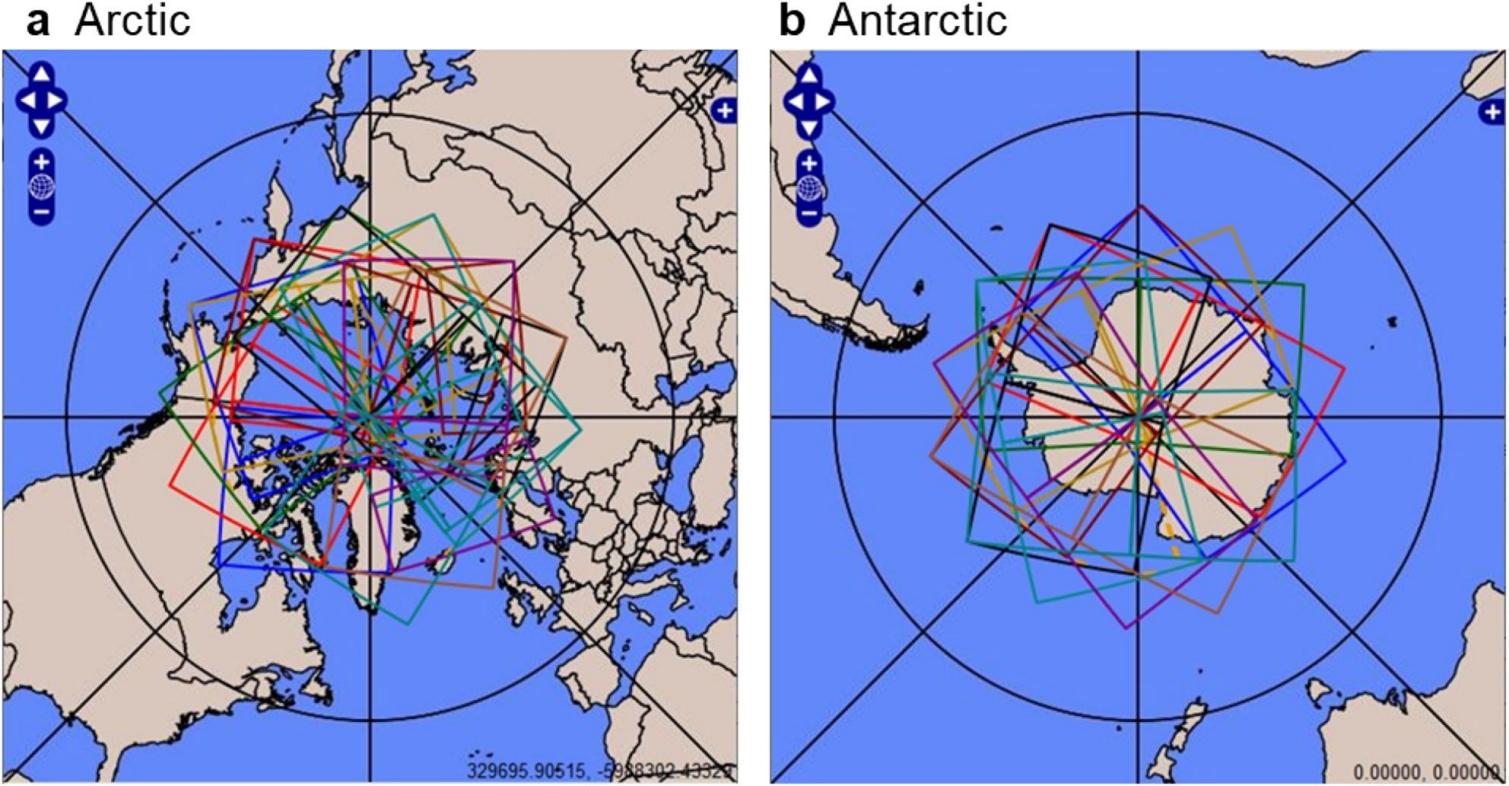
Area coverage for a typical 24-h period of the (**a**) Arctic and (**b**) Antarctic for the MetOp-A AVHRR. Overlap of swaths occur closer to the poles. (Image downloaded from the NOAA CLASS image search application.)

This study uses AVHRR imagery from the MetOp-A satellite with a nadir resolution of 1.1 km and swath width of approximately 2900 km. Data files were obtained on-line from the National Oceanic and Atmospheric Comprehensive Large Array-data Stewardship System. Data ranged from 01 June 2007 to 31 May 2019 and was constrained from 66° N to 90° N latitude for the Arctic and 66° S to 90° S latitude for the Antarctic. For the purposes of this research these latitude ranges are referred to as the Arctic and Antarctic regions. The polar orbit of the satellite allowed for 14 to 15 passes per day over the study areas, resulting in 127,996 images representing 2.24 × 10^11^ pixels. Table 1 shows specifications of the MetOp-A AVHRR sensor, while Table 2 lists statistics of analyzed data for each year of the study. Figure 1 illustrates the area coverage of a typical day for both poles.

**Table 1:** Selected specifications for the MetOp-A AVHRR sensor.

Parameter	Specification
Altitude/orbit type	827 km, sun synchronous, 14.2 orbits per day
AVHRR channel 1	0.58 to 0.68 μm (visible)
AVHRR channel 2	0.725 to 1.00 μm (visible/near infrared)
AVHRR channel 3a	1.58 to 1.64 μm (near infrared)
AVHRR channel 3b	3.55 to 3.93 μm (medium infrared)
AVHRR channel 4	10.30 to 11.30 μm (thermal infrared)
AVHRR channel 5	11.50 to 12.50 μm (thermal infrared)
File format	Full resolution area coverage
Spatial resolution	1.1 km at nadir degrading to approximately 8 km at swath edge
Sensor topology	Scanning ± 55° from nadir, 2900 km swath

**Table 2:** Statistics of analyzed data.

Year June—May	Arctic			Antarctic		
	Files	Size (GB)	Pixels	Files	Size (GB)	Pixels
2007–2008	5092	67.1	858,788,106	5083	65.6	9,083,412,480
2008–2009	5147	67.8	9,384,859,648	4975	64.2	8,888,973,312
2009–2010	4656	61.2	8,473,047,040	5178	66.9	9,256,345,600
2010–2011	5159	68.9	9,541,568,512	5186	67.1	9,278,058,496
2011–2012	6148	68.4	9,469,231,104	8205	95.6	13,226,854,400
2012–2013	6119	68.3	9,456,560,128	8603	97.3	13,459,331,072
2013–2014	4882	66.3	9,182,873,600	5171	66.8	9,236,019,200
2014–2015	5177	68.3	9,460,983,808	5181	66.9	9,260,939,264
2015–2016	5219	68.9	9,533,618,176	4976	64.0	8,857,276,416
2016–2017	5172	68.3	9,452,601,344	3755	47.7	6,605,512,704
2017–2018	4415	57.3	7,934,081,024	5186	67.2	9,296,461,824
2018–2019	4134	53.4	7,406,704,640	5177	67.3	9,313,036,288
Total	61,320	784.2	1.08 × 10^11^	66,676	836.6	1.16 × 10^11^

For this research, an algorithm was developed to perform bulk thermal calibration of Channel 4 (11 μm) and Channel 5 (12 μm) for each AVHRR file. Calibration procedures were in accordance with EUMETSAT Metop-A AVHRR specifications and the results compared against existing commercial software (L3Harris ENVI) for validity. BTD_11-12_ was determined for each pixel in a calibrated image and a threshold applied to determine the percentage of pixels with BTD_11-12_ < 0. The processed files were sorted by polar region and date, then sanitized for anomalies such as 100% dust coverage or extreme BTD_11-12_ values not supported by physics. A total of 583 files were removed (0.46%), resulting in a total of 127,413 images for the final analysis. The Python code for the algorithm used in the study is available on-line at https://github.com/matt-bowen/PyFRAC. The MetOp-A AVHRR is a stable sensor with calibration errors in the order of 0.3%^36^. Pixels close to the threshold may potentially be misclassified as dust or non-dust because of calibration errors, but statistically these should even out for the dataset.

In the polar environment over areas of snow/ice, water, and tundra where BTD_11–12_ > 0, suspended and deposited dust particulates return a distinctive negative value. Considering the underlying landscape, positive BTD_11-12_ values are still possible in the presence of dust since the pixel value is a summation of everything within the instantaneous field of view of the sensor. Space-based TIR radiometers such as AVHRR measure the skin temperature of an object, so only a thin layer of dust is necessary to produce a negative BTD_11-12_ signature. As such, the processed data reflects a dust concentration that is dense enough to dominate a pixel value. While it is possible that a substance other than mineral dust is causing large-scale negative BTD_11–12_ signatures in the polar environment, there is nothing in the literature to support this conjecture^30^. Thus, the method used in this study allows the detection of airborne and deposited dust in the Arctic and Antarctic with high confidence since the quantity of dust within a pixel must be sufficient to overcome the positive BTD_11-12_ contribution of the surface.

## Results

**Figure 2:**
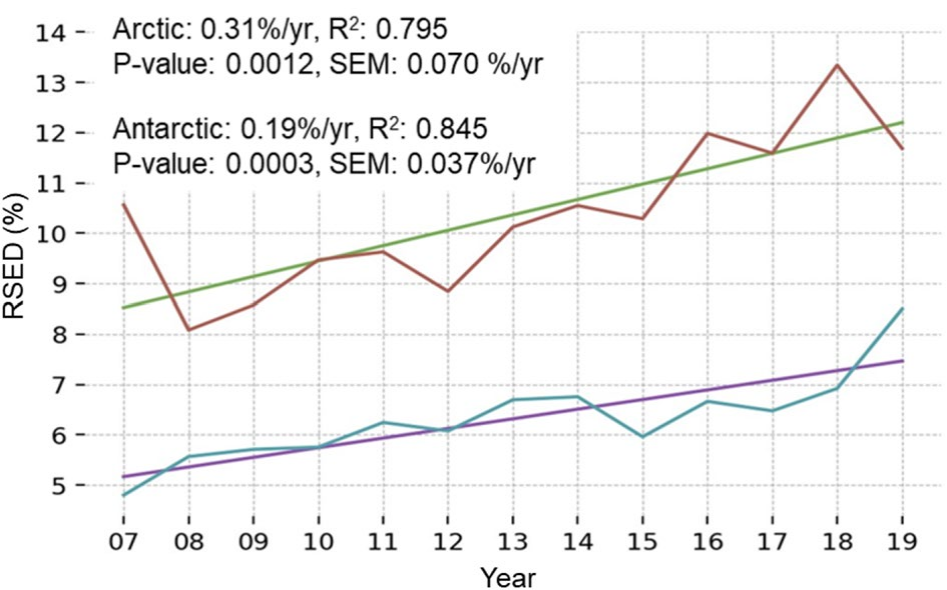
Yearly aggregated RSED for both polar regions between 2007 and 2019 with linear trends applied. The average dust extent increased by 0.31% and 0.19% per year for the Arctic and Antarctic, respectively. R^2^, P-Value and Standard Error of the Mean (SEM) indicate a strong linear correlation of the data.

The RSED is defined as the percentage of dust pixels (BTD_11-12_ < 0) in relation to the total number of pixels in a thermally calibrated AVHRR image. The yearly aggregated RSED showed strong linear trends for both poles between 2007 and 2019 (Fig. 2). In the Arctic, RSED increased from 8.5% to 12.1% or 0.31% per year, while in the Antarctic there was a smaller increase of 0.19% per year, ranging from 5.2 to 7.5%. The lower value in the southern polar region is reflective of decreased dust sources in that hemisphere.

**Figure 3:**
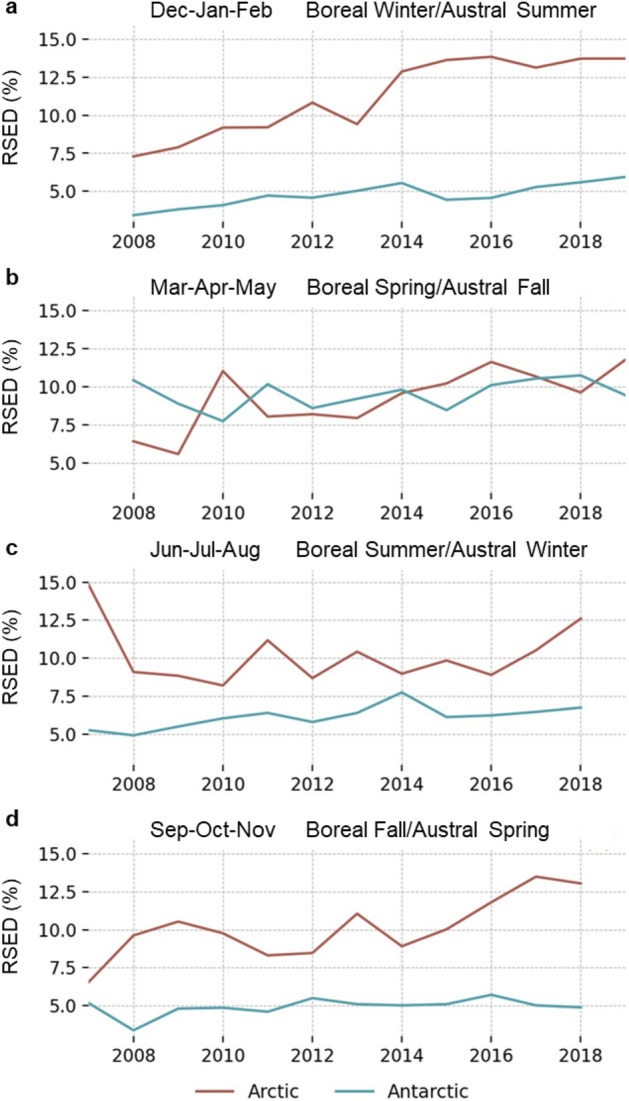
Seasonal RSED from 2007 to 2019 for (**a**) Dec–Jan–Feb, (**b**) Mar–Apr–May, (**c**) Jun–Jul–Aug, and (**d**) Sep–Oct–Nov for the Arctic (red) and Antarctic (blue).

Seasonal aggregated values for RSED were calculated for the study period. The boreal winter/austral summer is Dec–Jan–Feb followed by Mar–Apr–May (boreal spring/austral fall), Jun–Jul–Aug (boreal summer/austral winter), and Sep–Oct–Nov (boreal fall/austral spring). The dataset for this study begins in June 2007, which was the first available MetOp-A AVHHR imagery. The final season is Mar–Apr–May 2019, giving 12 years of seasonal data. Figure 3 shows the seasonal trends for both polar regions. The largest RSED rate increase occurred in Dec–Jan–Feb for both the Arctic (0.65% per year) and Antarctic (0.18% per year). The Arctic showed no change during the study period in boreal summer (Jun–Jul–Aug), while the Antarctic experienced minimal increase during the austral spring (Sep–Oct–Nov) and fall (Mar–Apr–May). The RSED rate increase for both poles were similar for Mar–Apr–May and features the only season when the average RSED of the Antarctic exceeded that of the Arctic. The average RSED for all seasons is markedly more stable for the southern pole region. Table 3 shows statistics for the seasonal RSED.

**Table 3:** Statistics for the seasonal RSED from 2007 to 2019 for each polar region, including average RSED and standard deviation (σ) as well as the linear trend and related R^2^, P-Value and Standard Error of the Mean (SEM).

Season 2007 to 2019	Average RSED (%)	σ (%)	Linear trend	R^2^	P-Value	SEM
**Arctic**						
Dec–Jan–Feb	11.2	2.5	0.65%/year	0.931	1.1030	0.081%/year
Mar–Apr–May	9.2	2.0	0.41%/year	0.745	0.0050	0.117%/year
Jun–Jul–Aug	10.2	1.9	− 0.03%/year	− 0.050	0.8767	0.169%/year
Sep–Oct–Nov	10.2	2.0	0.42%/year	0.758	0.0043	0.115%/year
**Antarctic**						
Dec–Jan–Feb	4.8	0.8	0.18%/year	0.852	0.0004	0.035%/year
Mar–Apr–May	9.5	1.0	0.09%/year	0.338	0.2821	0.078%/year
Jun–Jul–Aug	6.1	0.7	0.14%/year	0.691	0.0129	0.047%/year
Sep–Oct–Nov	4.9	0.6	0.07%/year	0.442	0.1500	0.045%/year

**Figure 4:**
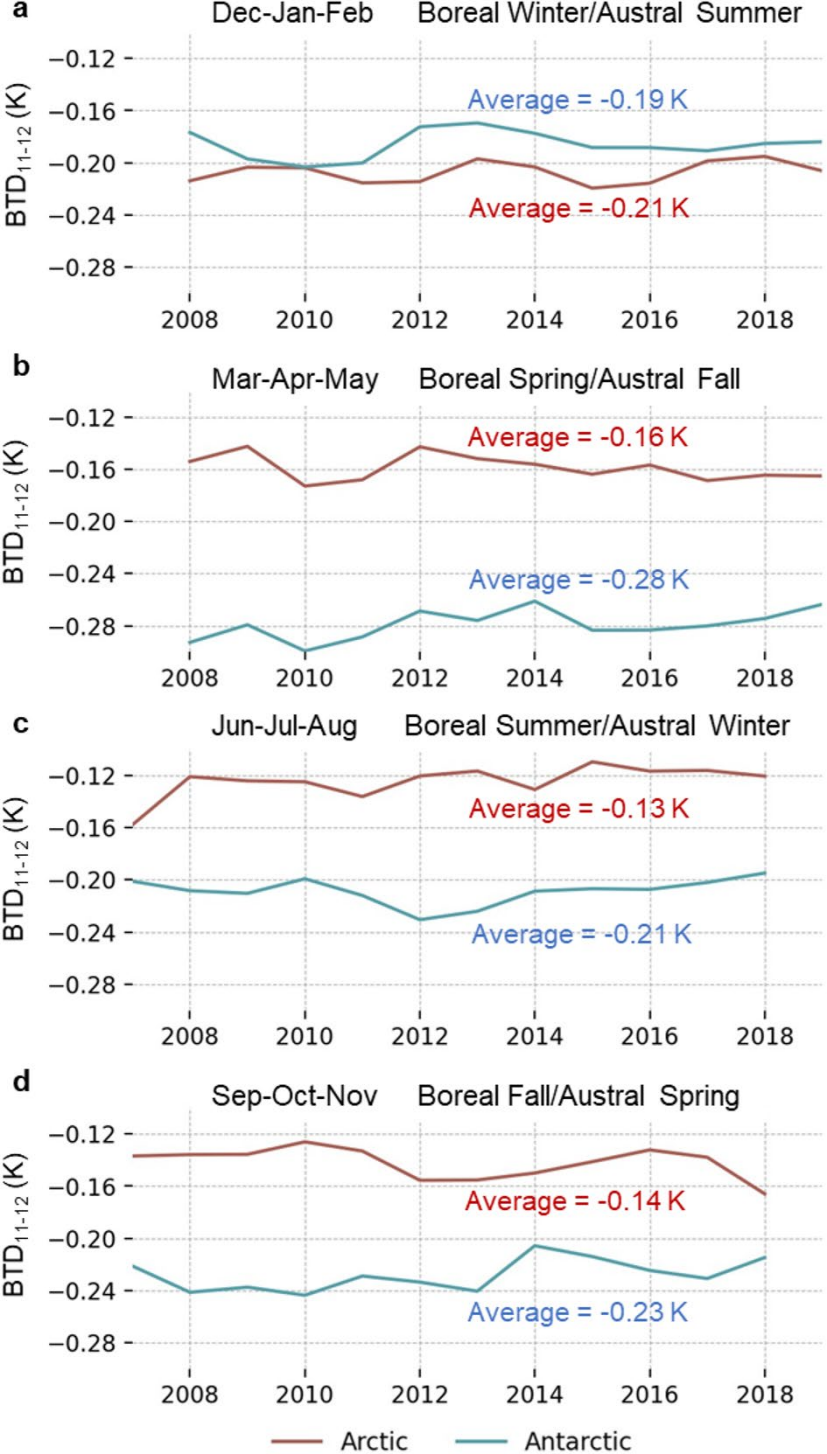
Seasonal BTD_11-12_ values from 2007 to 2019 for (**a**) Dec–Jan–Feb, (**b**) Mar–Apr–May, (**c**) Jun–Jul–Aug, and (**d**) Sep–Oct–Nov for the Arctic (red) and Antarctic (blue).

The mean value of BTD_11−12_ for all dust pixels was determined for each image. The overall average BTD_11−12_ was − 0.16 K for the Arctic and − 0.22 K for the Antarctic. Both regions showed consistent yearly averages with a standard deviation of 0.01 K for both poles. A seasonal analysis of BTD_11-12_ shows more variability (Fig. 4). There is a clear distinction between seasons in the Antarctic, with the most negative values occurring in Mar–Apr–May (− 0.28 K) and the least negative in Dec–Jan–Feb (− 0.19 K). The Arctic exhibits a smaller range of BTD_11-12_ values for most of the year (− 0.13 K to − 0.16 K) except for Dec-Jan-Feb when it reaches − 0.21 K. This is the only season when the average Arctic BTD_11-12_ is less than that of the Antarctic.

**Figure 5:**
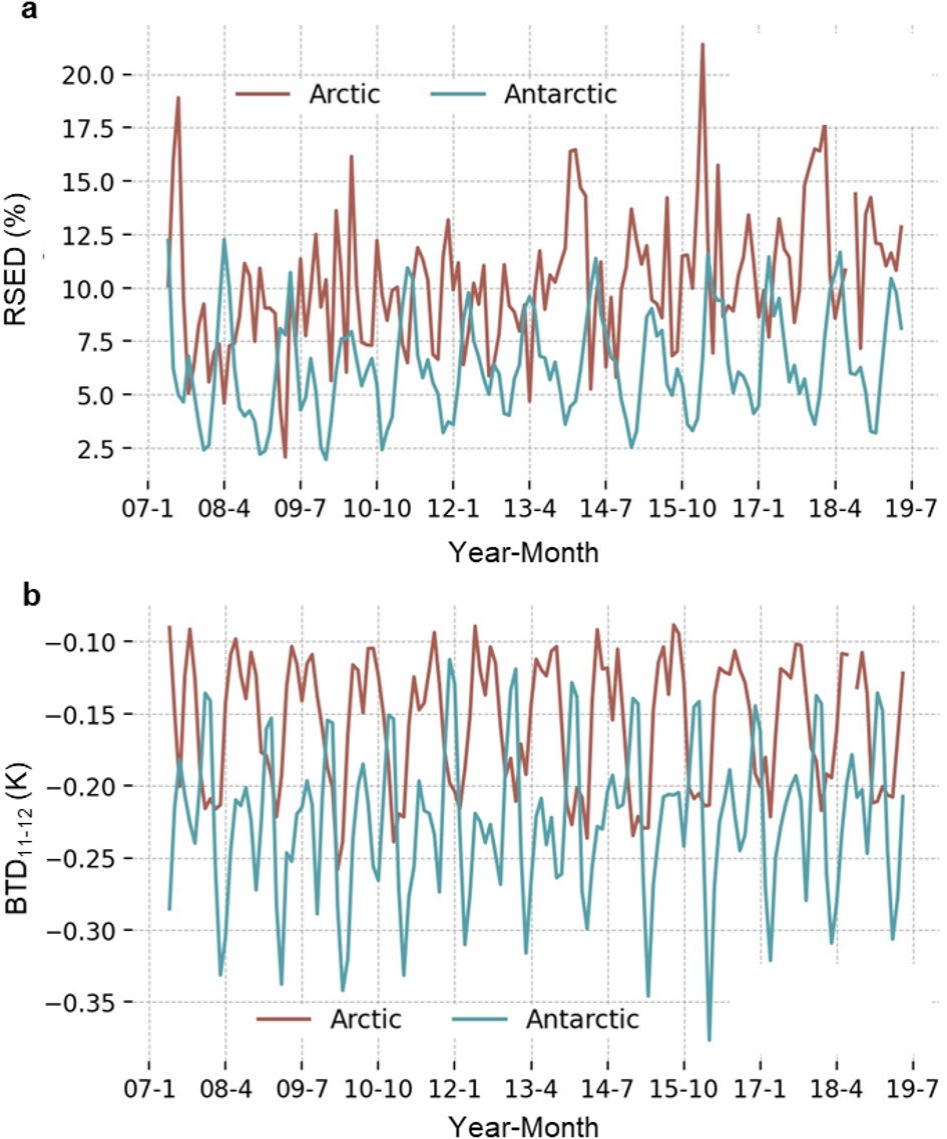
Monthly (**a**) RSED and (**b**) BTD_11−12_ for pixels containing dust from 2007 to 2019.

Finally, the results were aggregated monthly from June 2007 to May 2019. On a monthly basis the dataset becomes highly variable compared to the yearly and seasonal averages. When the two poles are compared, a pattern emerges with respect to RSED and BTD_11-12_ where peaks and troughs for both parameters are generally opposite between the two regions (Fig. 5). This overall view of the data is not surprising given the antipodal nature of the study areas.

## Discussion

### Annual trends

The principle result in this study indicates a yearly increase in average RSED rates at both poles (Fig. 2). This trend is likely a result of several factors, such as increased global dust generation, enhanced dust transport mechanisms and accelerating local dust production at high latitudes. Overall, the global amount of dryland has increased between 1948 and 2008, with this expansion expected to continue to the end of the twenty-first century^37^. Large-scale aridification can be attributed to many anthropogenic and natural variables^38^. The average aerosol optical depth (AOD) is a proxy for dust aerosol concentration and can be an indicator of transport mechanism strength. From 1998 to 2010, the AOD over the global oceans increased slightly, while stronger positive trends were associated with seasonal cycles^39^. Glacial activity is a contributor to local high latitude dust, as several processes surrounding glaciers are highly efficient at dust generation and emission. For example, strong winds are produced by gravity and thermal gradients associated with glaciers and the retreat of ice masses expose fine sediments for entrainment^40^. Dust produced by glaciers is expected to increase in the coming decades as glaciers throughout the cryosphere retreat^40^. In the Canadian Arctic Archipelago, nearly every glacier has shrunk since 1958, with region-wide retreat rates accelerating by a factor of five between 2000 and 2015^41^. The West Antarctic glaciers terminating in Pine Island Bay are also retreating rapidly. Smith and Kohler glaciers retreated more than 30 km between 1992 and 2011, with other glaciers in the area retreating between 9 and 14 km during the same time frame^42^.

The RSED in this research does not quantify the amount of dust transported to the poles but shows more widespread distribution since 2007. Modeling of polar dust loading indicates that 16 times more dust is transported to the Arctic than the Antarctic^16^, but the overall average RSED for the Arctic (10.4%, σ = 1.4% ) is only 4.1% greater than that of the Antarctic (6.3%, σ = 0.9%). Assuming that polar dust loading models are accurate, this implies that dust is spread more efficiently in the Antarctic. Dust transport mechanisms from global sources work on time scales of five days towards the Arctic^43^ and seven to ten days towards the Antarctic^44^. Finer particles are indicative of more distal sources^45^, which supports enhanced distribution of dust at the southern pole.

### Seasonal variations

Simulations indicate that dust loading in the Arctic peaks during boreal spring because of strengthening dust transport mechanisms from global sources^18^. In this study, Mar-Apr-May produced the lowest average Arctic RSED (9.2%, σ = 2.0%) with an increase of 0.41% per year. The maximum average Arctic RSED occurred during Dec-Jan-Feb (11.2%, σ = 2.5%) with the highest annual increase (0.62% per year) of any season. Total aerosol concentration in the Antarctic peaks in Dec–Jan–Feb^46,47^, which is concurrent with the highest increase in RSED rate (0.18% per year) but features the lowest observed average RSED (4.8%, σ = 0.8%). The results highlight the difference between total dust transport and spatial distribution. Finer particles spread more readily and may result in a higher RSED that is not a function of absolute dust quantity. This explains why RSED in this study does not necessarily align with established seasonal dust loading patterns.

### BTD_11-12_ variation

There is a notable contrast in the average value of BTD_11−12_ between the Arctic and Antarctic, with the southern polar region reporting a more negative signature except for Dec–Jan–Feb (Fig. 4). BTD_11-12_ generally decreases with decreasing particle size^48^. Larger dust particles are preferentially removed from atmospheric transport mechanisms over large distances^49,50^, suggesting that Arctic dust particles are potentially larger than the southern counterpart due to the more proximal sources in the northern hemisphere.

Another factor affecting BTD_11-12_ is dust composition. Mineral dust produces a negative BTD_11−12_ value because of the emissive properties of common silicate minerals present in desert sand^51^. Saharan and East Asian dust consist of 18.9% and 23.2% silicon respectively, while the primary Antarctic contributor of dust in Patagonia is 28.8% silicon with Australian dust at 18.5% silicon^52^. Since Patagonian and Australian dust affect different geographical areas of Antarctica, a detailed mapping of BTD_11-12_ values could help evaluate the relative importance of silicate concentration on BTD_11-12_ signatures.

A third influence on BTD_11-12_ signatures is the contribution of the underlying surface. The Antarctic continent and surrounding Southern Ocean consist almost entirely of water and ice throughout the year whereas the Arctic has a more varied surface, particularly in the warmer months. While the Antarctic surface makeup is relatively constant throughout the year with approximately 70% snow/ice and 30% water, the Arctic experiences seasonal fluctuations in the amount of ice, water, tundra, and bare rock. As such, there is more variation in BTD_11-12_ in the Arctic than the Antarctic. Interestingly, the most negative Arctic BTD_11-12_ occurs in Dec-Jan-Feb when the surface is almost entirely covered with snow/ice that has a highly positive BTD_11-12_. This implies that finer dust particles from more distal sources are present in the Arctic during boreal winter.

### Monthly variations

The monthly average RSED (Fig. 5a) shows a series of peaks and troughs for both poles, with the Arctic demonstrating greater variability (2.3% to 21.4%) than the Antarctic (2.1% to 12.5%). Long range transport for Arctic dust source regions such as the Taklamakan Desert vary on monthly scales^53^, while significant intermonth variations of dust deposition have been observed at Antarctic sites^23^. The RSED normally peaks around January in the Arctic and May in the Antarctic, while the troughs in each region generally correspond to the opposite’s peak. The greatest RSED in the Arctic occurred in February 2016 which coincided with the highest recorded value of the Oceanic Niño Index (ONI), suggesting a teleconnection with the El Niño-Southern Oscillation. Overall correlation between monthly Arctic RSED and ONI was 0.14 and slightly stronger for the North Atlantic Oscillation (0.18). The Antarctic showed no correlation with either climatic phenomenon, however, it is notable that the lowest average BTD_11-12_ occurred in March 2016 during the period of record high ONI.

Monthly BTD_11-12_ (Fig. 5b) show greater monthly variation in the Antarctic (− 0.38 K to − 0.12 K) than the Arctic (− 0.26 K to − 0.09 K). This may be attributable to finer dust in the Antarctic causing low BTD_11-12_ values that is offset periodically by reduced dust concentration. A strong negative correlation (− 0.67) was revealed between BTD_11-12_ and RSED in the Arctic with a moderate negative correlation (− 0.30) in the Antarctic. The negative correlation indicates that there is greater areal spreading of dust as BTD_11-12_ decreases, implying that finer dust particles are spread more readily. This relationship is pronounced in the Arctic winter where the highest seasonal RSED and lowest seasonal BTD_11-12_ both occur in Dec–Jan–Feb, which coincides with the strongest boreal surface winds^54^.

### Significance of study

This research offers unique insight on recent trends in polar dust extent using satellite thermal infrared imagery. Whereas the results show an increase in the spatial extent of dust at both poles, there are variations between the two areas that suggest different mechanisms of dispersal and distinct dust characteristics. Modelled seasonal dust loading peaks do not generally align with the maximum spreading of dust, which may have significant impact on climate models with respect to the darkening of polar surfaces and the influence on cloud radiative properties. The next step in this research is to map areas of Arctic and Antarctic dust influx.

## Data Availability

Satellite Advanced Very High Resolution (AVHRR) data is available on-line at the National Oceanic and Atmospheric Administration (NOAA) Comprehensive Large Array-data Stewardship System (CLASS) (https://www.bou.class.noaa.gov/saa/products/welcome).
